# Association between Shock Index and Emergency Department Cardiac Arrest

**DOI:** 10.1155/2021/9138449

**Published:** 2021-10-25

**Authors:** Chao-Tung Chen, Pei-Ming Wang, Chao-Hsin Wu, Chih-Wei Wei, Tai-Lin Huang

**Affiliations:** ^1^Department of Family Medicine, Kaohsiung Chang Gung Memorial Hospital, Chang Gung University College of Medicine, Kaohsiung, Taiwan; ^2^Emergency Center of Tungs' Taichung MetroHarbor Hospital, Taichung, Taiwan

## Abstract

**Background:**

In the emergency department (ED), early identification of patients at risk of cardiac arrest is paramount, especially in the context of overcrowding. The shock index (SI) is defined as the ratio of heart rate to systolic blood pressure. It is a tool used for predicting the prognosis of critically ill and injured patients. In this study, we have discussed the relationship between SI and cardiac arrest in the ED.

**Methods:**

Patients who experienced cardiac arrest in the ED were classified into two groups, SI ≥ 0.9 and < 0.9, according to their triage vital signs. The association between SI ≥ 0.9 and in-hospital mortality was analyzed in five different etiologies of cardiac arrest, including hypoxia, cardiac cause, bleeding, sepsis, and other metabolic problems.

**Results:**

In total, 3,313 patients experienced cardiac arrest in the ED. Among them, 1,909 (57.6%) had a SI of ≥0.9. The incidence of SI ≥ 0.9 in the five etiologies was 43.5% (hypoxia), 58.1% (cardiac cause), 56.1% (bleeding), 58.0% (sepsis), and 65.5% (other metabolic problems). SI was associated with in-hospital mortality (adjusted odds ratio (aOR), 1.6; 95% confidence interval (CI), 1.5–1.8). The aOR (CI) in the five etiologies was 1.3 (1.1–1.6) for hypoxia, 1.8 (1.6–2.1) for cardiac cause, 1.3 (0.98–1.7) for bleeding, 1.3 (1.03–1.6) for sepsis, and 1.9 (1.5–2.1) for other metabolic problems.

**Conclusion:**

More than half of the patients who experienced cardiac arrest in the ED had a SI ≥ 0.9. The SI was also associated with in-hospital mortality after cardiac arrest in the ED. SI maybe used as a screening tool to identify patients at risk of cardiac arrest in the ED and a predictor of mortality in those experiencing cardiac arrest in the ED.

## 1. Introduction

Cardiac arrests in the emergency department (ED) can be categorized as out-of-hospital cardiac arrests (OHCAs) and cardiac arrests in the ED. Patients experiencing cardiac arrest in the ED potentially have a higher proportion of reversible etiologies and neurologically intact survival than those experiencing out‐of‐hospital cardiac arrest transported to the ED [1, 2]. Early or late detection of cardiac arrest can determine the prognosis. However, it is difficult for emergency physicians to recognize patient deterioration due to crowding in the ED caused by the long boarding times for inpatient and intensive care unit beds [3–6]. The shock index (SI), defined as the ratio of heart rate to systolic blood pressure (SBP), is a tool used for predicting the prognosis of acutely ill and injured patients [7]. It has been used to predict outcomes in patients with severe sepsis [8, 9], hemorrhagic shock [10], pulmonary embolism [11], and acute myocardial infarction [12]. Maheshwari et al. used a single SI reading of ≥0.9 as a predictor of mortality among critically ill patients [13]. However, few studies have used SI for predicting cardiac arrest in the ED. In this study, we analyzed the association between SI and the prognosis of patients experiencing cardiac arrest in the ED. We assumed that patients experiencing ED cardiac arrests had a high incidence of SI ≥ 0.9 on ED arrival and that these patients had unfavorable prognoses after cardiac arrest.

## 2. Materials and Methods

### 2.1. Study Setting

Data were obtained from a large healthcare system in Taiwan, including two tertiary referral medical centers and two secondary regional hospitals. The cumulative mean annual number of ED visits in the study settings was approximately 450,000 visits per year. All patient records and information were anonymized and deidentified prior to analysis. This retrospective study was approved by the Institutional Review Board (IRB) of Chang Gung Medical Foundation (IRB No. 202101531B0).

### 2.2. Study Participants

From January 2010 to December 2016, patients aged >17 years with nontraumatic disease who were experiencing cardiac arrest, thereby requiring cardiopulmonary resuscitation in the ED, were included in the study. Patients experiencing OHCA and those with do-not-resuscitate orders were excluded.

### 2.3. Measurements

Data on patient demographics and medical history were extracted from electronic health records. Patients were categorized into five groups according to the etiologies of cardiac arrest—hypoxia, cardiac cause, bleeding, sepsis, and other metabolic problems. Diagnosis was based on the diagnostic codes from the International Classification of Diseases, Ninth and Tenth Revision, Clinical Modification. Patients' triage vital signs (heart rate and blood pressure) were used to calculate SI. SI was calculated by dividing the heart rate by SBP. In-hospital 30-day mortality was selected as the outcome. The association between SI ≥ 0.9 and mortality was analyzed [13]. In addition, to determine the advantage of SI, patients were further grouped according to SBP <90 mmHg and ≥90 mmHg. The association between SBP <90 mmHg and mortality was also analyzed.

### 2.4. Data Analysis

Regarding continuous variables, age was presented as mean ± standard deviation. Vital signs, including body temperature, heart rate, blood pressure, and blood oxygen saturation, were presented as median ± quartile deviation. The distributions of categorical data were presented as numbers and percentages. Student's *t*-test, Mann–Whitney *U* test, and chi-square test were used to conduct the analysis. The difference in survival duration between patients with SI ≥ 0.9 and SI < 0.9 and those with SBP <90 mmHg and ≥90 mmHg was assessed using Kaplan–Meier analysis.

To determine the associations between mortality and SI ≥ 0.9 and SBP <90 mmHg, Cox regression analyses were performed. The effects were estimated using hazard ratios and corresponding 95% confidence intervals (CIs). Results were considered statistically significant for two-tailed tests at *P* < 0.05. The IBM Statistical Package for the Social Sciences for Windows version 22.0 (released 2013, IBM Corp., Armonk, NY, USA) was used for all statistical analyses.

## 3. Results

**Figure 1:**
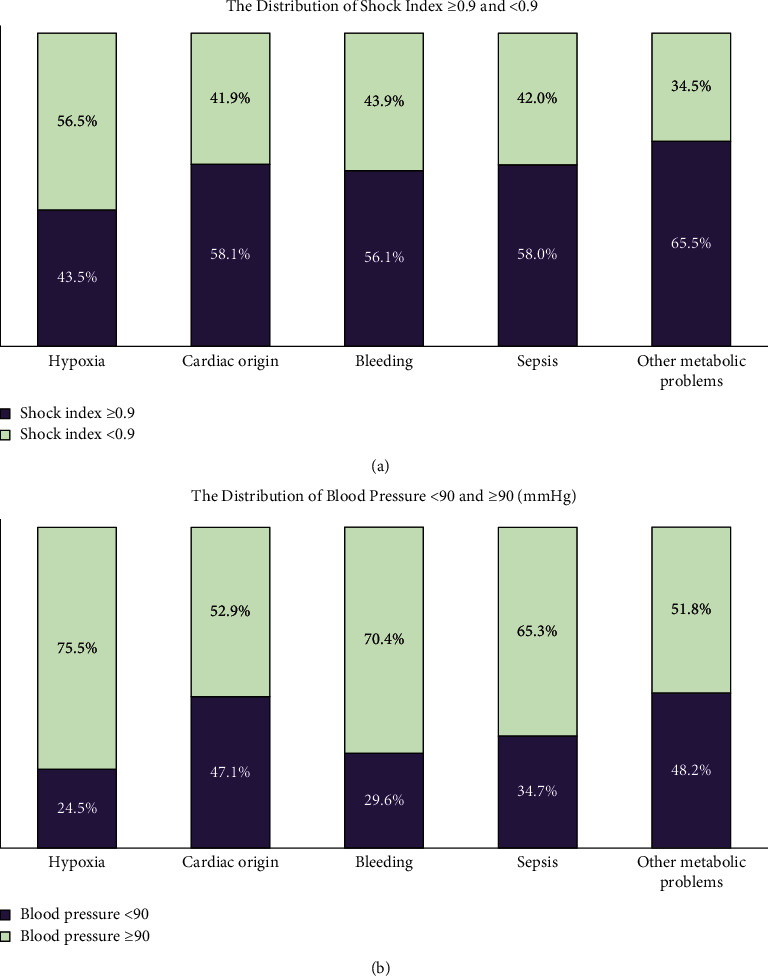
(a) Distribution of shock index ≥0.9 and <0.9 in the five etiologies of cardiac arrest in the emergency department. (b) Distribution of systolic blood pressure <90 mmHg and ≥90 mmHg in the five etiologies of cardiac arrest in the emergency department.

In total, 3,313 nontrauma patients experienced cardiac arrest in the ED. Among them, 1,909 (57.6%) had a SI of ≥0.9 and 1,348 (40.7%) had a SBP of <90 mmHg. The demographic characteristics of the patients are given in Table 1. The distribution of SI ≥ 0.9 and SI < 0.9 in the five etiologies is shown in Figure 1(a). Patients experiencing cardiac arrest due to hypoxia had the lowest incidence of SI ≥ 0.9, followed by those experiencing cardiac arrest due to bleeding, sepsis, cardiac cause, and other metabolic problems. The distributions of SBP <90 mmHg and SBP ≥90 mmHg are shown in Figure 1(b). Patients experiencing cardiac arrest due to cardiac and other metabolic problems had a higher incidence of SPB <90 mmHg than those experiencing cardiac arrest due to the other three etiologies.

**Figure 2:**
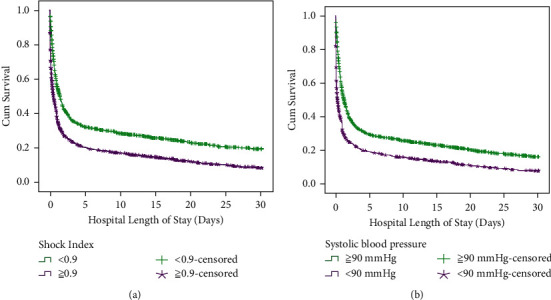
(a) Survival analysis of patients with shock index ≥0.9 and <0.9. (b) Survival analysis of patients with systolic blood pressure <90 mmHg and ≥90 mmHg.

Figures 2(a) and 2(b) show the results of the Kaplan–Meier survival analysis. Patients with SI ≥ 0.9 and SBP <90 mmHg had shorter survival duration than those with SI < 0.9 and SBP ≥90 mmHg. To determine the association between mortality and SI and SBP, logistic regression analysis was performed. After controlling for age and sex, SI and SBP were associated with in-hospital 30-day mortality. Stratified analysis according to different etiologies of cardiac arrest was also conducted. SI ≥ 0.9 was associated with in-hospital 30-day mortality in patients experiencing cardiac arrest due to hypoxia, cardiac cause, sepsis, and other metabolic problems, while SBP <90 mmHg was associated with in-hospital 30-day mortality in patients experiencing cardiac arrest due to cardiac cause and other metabolic problems (Table 2).

## 4. Discussion

Crowding in the ED is a global issue as patients remain in the ED for a long time either for assessment or pending admission [14]. In the past few decades, studies have reported the association between ED crowding and unfavorable outcomes [15–19]. As patients continue to spend more time in the ED, timely detection of patient deterioration is critical. However, patient deterioration in the ED is hardly recognized due to the variable frequency of vital sign observations [3–6]. Therefore, it is important to develop early warning scores to predict and prevent cardiac arrest in the ED for this vulnerable population [20]. In our study, 57.6% of the patients experiencing cardiac arrest in the ED had a SI of ≥0.9, while 40.7% of patients had a SPB of<90 mmHg. This difference in the incidence was more prominent after the stratified analysis. Therefore, SI can be used as a simple criterion to identify high-risk patients. In a recent study, SI during ED stay correlated with in-hospital mortality and early mortality in patients admitted to the intensive care unit via the ED [21]. Since patients have prolonged stay in the ED, especially amidst the COVID-19 pandemic, some cardiac arrests that usually occur after hospital admission may occur in the ED. Therefore, using SI to predict and prevent cardiac arrest in the ED seems to be a reasonable strategy.

The incidence of SI ≥ 0.9 and SBP <90 mmHg was different in distinct etiologies of cardiac arrest in the ED. While the incidence of SI ≥ 0.9 was over 56% in most etiologies, the incidence of SBP <90 mmHg was higher than 47.1% only when the etiologies were cardiac cause and other metabolic problems. The rest of the etiologies had an incidence of less than 34.7%. Therefore, as a tool for predicting cardiac arrest in high-risk patients, SI ≥ 0.9 is more predictive than SBP <90 mmHg. The incidence of SI ≥ 0.9 in cardiac arrest due to hypoxia was only 43.5%, which might be because SI is designed to provide an approximation of hemodynamic status in addition to traditional vital signs [7]. Hemodynamic changes may not be present in the early stage of hypoxia. For example, patients with chronic obstructive pulmonary disease visit the ED for hypoxia. These patients may have tachycardia accompanied with hypertension; thus, the SI of these patients may be normal. Therefore, application of SI in patients with hypoxia should be carefully considered.

SI ≥ 0.9 and SBP <90 mmHg are also associated with the prognosis of cardiac arrest in the ED. In the survival analysis of our study, the in-hospital 30-day mortality rate of patients with SI ≥ 0.9 was higher than that of patients with SI < 0.9. A similar finding was noted for patients with SBP <90 mmHg and ≥90 mmHg. There was an association between mortality and SI < 0.9 and SBP <90 mmHg. However, in the stratified analysis, SI ≥ 0.9 was associated with mortality in patients experiencing cardiac arrest due to hypoxia, cardiac cause, sepsis, and other metabolic problems, while SBP <90 mmHg was associated with cardiac arrest due to cardiac cause and other metabolic problems. Therefore, SI ≥ 0.9 might be a better predictor of mortality than SBP <90 mmHg. Previous studies have used SI to predict the outcomes of patients with severe sepsis [8, 9], hemorrhagic shock [10], pulmonary embolism [11], and acute myocardial infarction [12]. These diagnoses are often related to cardiac arrest, and the conclusions of these studies are similar to those of our study. Therefore, SI may be used both as a criterion to define high-risk patients and a predictor of mortality due to cardiac arrest in the ED.

## 5. Limitations

There are some limitations to this study. First, the retrospective nature of this database study made it difficult to collect data and determine the precise etiology of cardiac arrest. Patient data were anonymized and deidentified before analysis; hence, chart reviews were also infeasible. In addition, the diagnostic codes inputted by ED physicians might have been less precise because of limitations in time and availability of clinical information. Therefore, misclassification of the etiologies of cardiac arrest may have occurred. Second, demographic records were limited for some patients who died in the ED. Therefore, data on some confounding factors, such as comorbidities, were missing; hence, in the logistic regression model, we did not adjust for these confounding factors. Finally, we only considered triage vital signs. However, some patients had prolonged ED boarding; thus, the use of triage SI in these patients might be less appropriate. We believe that if a vital sign is identified earlier in a certain period, cardiac arrest can be predicted more precisely. However, further studies are needed to confirm this.

## 6. Conclusions

More than half of the patients experiencing cardiac arrest in the ED had a SI of ≥0.9. SI was also associated with mortality after cardiac arrest in the ED. SI maybe used as a screening tool for patients at risk of cardiac arrest and as a predictor of mortality after cardiac arrest in the ED.

## Figures and Tables

**Table 1:** Demographics of patients who developed cardiac arrest in the emergency department.

Shock index	*n* = 3,313
Age	67.4 ± 15.7
Male	2,102 (63.4%)
Vital sign at triage	
Body temperature (°C)	36.2 ± 0.8
Heart rate (times/min)	101 ± 20
Systolic blood pressure (mmHg)	120 ± 26
Diastolic blood pressure (mmHg)	72 ± 16
Oxygen saturation (%)	93 ± 7
Shock index ≥0.9	1,909 (57.6%)
Systolic blood pressure <90 mmHg	1,348 (40.7%)
Etiology of cardiac arrest	
Hypoxia	547 (16.5%)
Cardiac cause	1120 (33.8%)
Bleeding	253 (7.6%)
Sepsis	452 (13.6%)
Other metabolic problem	941 (28.4%)
In-hospital mortality	2,585 (78.0%)

**Table 2:** The association between in-hospital 30-day mortality and shock index ≥0.9 and blood pressure <90 mmHg in different etiologies of cardiac arrest.

	Shock index ≥0.9		Systolic blood pressure <90 mmHg	
Etiology	aOR	95% CI of OR	aOR	95% CI of OR
All	1.6^*∗*^	1.5–1.8	1.7^*∗*^	1.6–1.9
Hypoxia	1.3^*∗*^	1.1–1.6	1.2	1.0–1.6
Cardiac cause	1.8^*∗*^	1.6–2.1	1.8^*∗*^	1.6–2.1
Bleeding	1.3	1.0–1.7	1.4	1.0–1.9
Sepsis	1.3^*∗*^	1.03–1.6	1.2	1.0–1.5
Other metabolic problem	1.8^*∗*^	1.5–2.1	1.9^*∗*^	1.7–2.2

OR, odds ratio; CI, confidence interval; aOR, adjust for age and sex by COX regression. ^*∗*^Statistic significance.

## Data Availability

The data of this study are supported by the Chang Gung Memorial Hospital and cannot be shared due to the policy of the study setting.
